# Human Oral Mucosa as a Potentially Effective Source of Neural Crest Stem Cells for Clinical Practice

**DOI:** 10.3390/cells12182216

**Published:** 2023-09-06

**Authors:** Fawzia Bardag Gorce, Mais Al Dahan, Kavita Narwani, Jesus Terrazas, Monica Ferrini, Colonya C. Calhoun, Jettie Uyanne, Jun Royce-Flores, Eric Crum, Yutaka Niihara

**Affiliations:** 1 The Lundquist Institute for Biomedical Innovation, Torrance, CA 90502, USAyniihara@emmauslifesciences.com (Y.N.); 2 Division of Oral & Maxillofacial Surgery and Hospital Dentistry, Department of Surgery Harbor UCLA Medical Center, Torrance, CA 90502, USA; 3 Charles R. Drew University of Medicine and Science, Los Angeles, CA 90059, USA; 4 Department of Surgery, UCLA, David Geffen School of Medicine, Los Angeles, CA 90095, USA; 5 UCLA School of Dentistry, Los Angeles, CA 90095, USA; 6 Department of Oral & Maxillofacial Surgery and Hospital Dentistry, University of Michigan School of Dentistry, Ann Arbor, MI 48109, USA; 7 Herman Ostrow School of Dentistry of USC, Los Angeles, CA 90089, USA; 8 Emmaus Medical, Inc., Torrance, CA 90503, USA

**Keywords:** oral mucosa epithelium, neural crest stem cells, KaFa medium

## Abstract

We report in this study on the isolation and expansion of neural crest stem cells (NCSCs) from the epithelium of oral mucosa (OM) using reagents that are GMP-certified and FDA-approved for clinical use. Characterization analysis showed that the levels of keratins *K2*, *K6C*, *K4*, *K13*, *K31*, and *K15*—specific to OM epithelial cells—were significantly lower in the experimental NCSCs. While *SOX10* was decreased with no statistically significant difference, the earliest neural crest specifier genes *SNAI1/2*, *Ap2a*, *Ap2c*, *SOX9*, *SOX30*, *Pax3*, and *Twist1* showed a trend in increased expression in NCSCs. In addition, proteins of *Oct4*, *Nestin* and *Noth1* were found to be greatly expressed, confirming NCSC multipotency. In conclusion, our study showed that the epithelium of OM contains NCSCs that can be isolated and expanded with clinical-grade reagents to supply the demand for multipotent cells required for clinical applications in regenerative medicine. Supported by Emmaus Medical Inc.

## 1. Introduction

The oral mucosa (OM) shows the essential quality of quick regeneration [1] and a slower rate of ageing in comparison to other tissues in the body [2]. Both the epithelia and the lamina propria of OM contain stem cells that have been shown to be good candidates for stem cell therapies, tissue engineering and regenerative medicine [1,2]. OM represents an on-demand easy access source of autologous cells and is a rich source of a large number of stem cells. OM stem cells (OMSCs) are a significant source of stem cells and have the capacity to differentiate into cell lineages outside their tissue of origin to regenerate corneal epithelium [3], burned skin [4], and the esophagus [5]. OM lamina propria contains mesenchymal stem cell populations that can be differentiated to form bone cells [6,7,8,9] or neuronal–dopaminergic cells with therapeutic potential in the hemi-Parkinsonian rat model [10] or differentiated into astrocyte-like cells that provide peripheral neuroprotection, significantly increasing motor performance after sciatic nerve injury in rats [11]. Using OMSCs, wound healing was enhanced in a diabetic mouse model [12], anastomotic leak rates were reduced, and improved postoperative wellness in a murine model of colon surgery was noticed [13]. A 3D construct of OMSCs was engineered and implanted and achieved substantial recovery in spinal cord injury [14]. Developmental biology studies state that craniofacial structures and oral cavity tissues contain neural crest stem cells (NCSCs) [15]. NCSCs can be differentiated into various cell types, which makes them a valuable cell source for regenerative medicine. NCSCs, specified from the ectoderm, are migratory multipotent progenitors that have the developmental potential to differentiate into a variety of diverse cell types, including peripheral neurons, bone and cartilage, and melanocytes [16]. NCSCs can also differentiate into both ectodermal derivatives (neurons and glia) and ectomesenchymal derivatives (chondrocytes and osteocytes) [17].

However, current established NCSC isolation and expansion protocols are currently still utilizing xenogeneic products that are not approved by U.S regulatory agencies for clinical applications. Isolating and expanding NCSCs with clinical-grade reagents and following good manufacturing practice (GMP) guidelines will be required for a safe cell-based therapy [18]. Only FDA-approved and GMP-grade cell culture reagents that are free from animal-derived growth supplements will lead to OM-derived NCSCs approved for clinical application.

In the present study, we describe the isolation and expansion of NCSCs from a small biopsy of the epithelium of human buccal tissue. The cell expansion was accomplished using KaFa medium that was free of fetal bovine serum and contained supplements certified for clinical application [18]. Only recently, it was reported that stem cells had been isolated from a small biopsy of the gingiva, and cells were expanded in a new serum-free medium and differentiated into bone cells [19]. Our techniques avoid the need to extract a tooth to obtain the dental pulp stem cells or to cut the gingiva or even to use induced pluripotent stem cells (iPSCs). Under local anesthesia, a small biopsy of oral mucosa epithelium allows for the isolation and expansion of NCSCs that can be banked for further autologous regenerative grafting. The expansion of NCSCs using clinical-grade cell culture conditions will speed up the process of developing NCSC-based therapies in regenerative medicine.

## 2. Material and Methods

### 2.1. Setting of Human Research

The involvement of human participants was in accordance with the ethical standards of the institutional and national research committee and with the 1964 Helsinki Declaration and its later amendments or comparable ethical standards. The study was reviewed and approved by the John F. Wolf, M.D. Human Subjects Committee (Institutional Research Board Committee, IRB) of the Lundquist Institute.

Buccal tissue biopsies were obtained from donors following study approval. Healthy volunteers, age 18 or older, were recruited. After informed consent was obtained from study participants, procedures were performed by a surgeon in the Division of Oral and Maxillofacial Surgery Clinic at Harbor UCLA Medical Center.

Using local anesthesia, a thin surface “epithelial shaving” was performed in the buccal mucosa (cheek), just above the occlusal plane, avoiding the parotid duct, as progenitor stem cells are more concentrated in this area. (See following figure).

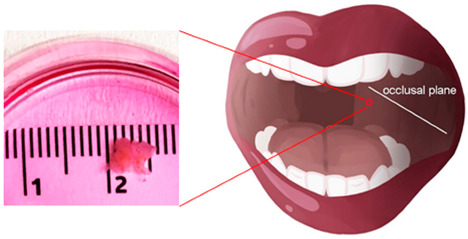



Biopsy of buccal mucosa tissue: A sterile dermal punch 6 mm in diameter was used to create a uniform demarcation of the area to be excised (red circle). Circular pieces of tissue of approximately 2 mm in depth were removed using dissecting scissors and tissue forceps grasping the demarcated mucosa.

The excised wound was sutured using 4-0 chromic sutures. The excised tissues were immediately immersed in high-glucose basal medium KODMEMF12 to sustain cell viability. The de-identified specimen was transported to a cell culture room, immersed in the basal medium KODMEMF12 in a primary receptacle (a tube with screw-top lids to prevent accidental opening), and placed vertically in a transportation box maintained at 4 °C.

### 2.2. Cell Isolation and Culture

KaFa medium was prepared as follows: the Cell Therapy Systems (CTS) product line of Thermo Fisher Scientific Inc. was used as it is manufactured in conformity with cGMP for medical devices, 21 CFR Part 820 USP<1043> and Ph Eur 5.2.12 (https://www.thermofisher.com/us/en/home/clinical/cell-gene-therapy/cell-therapy/cell-therapy-systems.html). CTS products are designed to reduce the burden in qualifying reagents for clinical applications. The components used in the formulation of KaFa medium are described in our patent LAB0223_2021-11-04_0WVR-305372-WO, Serial No.: PCT/US2021/058042. Briefly, the main components include CTS KO ™ DMEM/F-12 (ThermoFisher Scientific, Waltham, MA, USA), KO serum replacement (ThermoFisher Scientific, Waltham, MA, USA), Albutein (GRIFOLS, Los Angeles, CA, USA), D-glucose (ThermoFisher Scientific, Waltham, MA, USA), B27 (ThermoFisher Scientific, Waltham, MA, USA), GlutaMAX™ CTS Supplement (ThermoFisher Scientific, Waltham, MA, USA), Sodium Pyruvate (ThermoFisher Scientific, Waltham, MA, USA), SOLU-CORTEF (Mckesson, Santa Fe, CA, USA) Triostat (3,3′,5-Triiodo-L-thyronine sodium salt) (X Gen Pharmaceuticals Inc., Horseheads, NY, USA), Antibiotic-Antimycotic (ThermoFisher Scientific, Waltham, MA, USA), Isoproterenol Hydrochloride (Nexus Pharmaceuticals Inc., Lincolnshire, IL, USA), ITS (Insulin, Transferin, Selenium) (ThermoFisher Scientific, Waltham, MA, USA), EGF (R&D system, Minneapolis, MN, USA), and ROCK Inhibitor (&D system, Minneapolis, MN, USA). Once the medium was prepared, it was stored at 4 °C in a dark room and could be used to feed cells within 1 month.

The biopsied buccal tissue was cut into smaller pieces that were incubated with Dispase^®^ I (neutral protease, grade I. Roche Diagnostics GmbH, Mannheim, Germany) for 1 h at 37 °C, which allowed the epithelium to be separated from the lamina propria. Epithelium pieces were subjected to trypsin (TrypLE^TM^, ThermoFisher Scientific, Waltham, MA, USA) digestion to extract and isolate epithelial cells. Trypsin digestion was inactivated using our newly designed clinical-grade KaFa medium. After centrifugation, isolated cells were re-suspended in a small volume of KaFa medium and were seeded at a density of 0.1–0.3 × 10^5^ cells/cm^2^. Cells were cultured for about 3 weeks at 37 °C in a humidified atmosphere containing 5% CO_2_. KaFa medium replacement was scheduled every other day in week 1, then every day in weeks 2 to 3. The freshly isolated and seeded cells were at passage zero (P0). After 2 to 3 weeks, the cells formed colonies that grew and expanded to reach about 60% confluency. Cell colonies were transferred (passage 1 (P1)) using trypsin digestion and reseeded to grow into a confluent monolayer cell sheet. Microscopic analysis and imaging of the successfully expanded cells was performed before cell harvesting. P1 cells were then collected using a cell scraper. Cultured cells were washed three times with PBS, scraped off the cell culture ware, and transferred to an Eppendorf tube for subsequent analysis.

### 2.3. RNA Sequencing

#### 2.3.1. Library Construction and Sequencing

Libraries for RNA-Seq were prepared with the KAPA Stranded mRNA-Seq Kit. The workflow consisted of mRNA enrichment and fragmentation, first-strand cDNA synthesis using random priming followed by second-strand synthesis converting cDNA: RNA hybrid to double-stranded cDNA (dscDNA), and the incorporation of dUTP into the second cDNA strand.

cDNA generation was followed by end repair to generate blunt ends, A-tailing, adaptor ligation, and PCR amplification. Different adaptors were used for multiplexing samples in one lane. Sequencing was performed on Illumina HiSeq3000 for a SE 1 × 50 run. Data quality checks were performed on Illumina SAV. Sample de-multiplexing was performed with Illumina bcl2fastq v2.19.1.403 software.

#### 2.3.2. Bioinformatics and Data Analysis

Reads were mapped with STAR 2.7.9a [20]. Read counts per gene were quantified using a human Ensembl GRCh38.104 GTF file. In Partek Flow [21], read counts were normalized via CPM + 1.0 × 10^−4^. The investigated genes in the present study were keratin genes (*K2*, K6C, K4, K13, K31, and K15) specific to oral mucosa epithelial cells and genes that specific to NCSCs (*Snai2*, *SOX9*, *SOX30*, *Pax3*, *OCT-4*, and *Nothc1/2*).

All results of differential gene expression analysis utilized the statistical analysis tool DESeq2 [22]. Filters were applied to differentially expressed gene lists, *p*-values, FDR, and fold change (FC). The filter was *p* < 0.001, FDR < 0.01 and FC > 2-fold for all differential gene expression results. QIAGEN Ingenuity Pathway Analysis software (IPA) [23] was used to perform pathway analysis.

Using the list of significantly differentially expressed genes, canonical pathway analysis, disease and function analysis, and network analysis were performed in IPA. Investigated genes included keratin genes (K4, K13, and K15) that are specific to oral mucosa epithelial cells and genes that are specific to NCSCs (*Snai2*, *SOX9*, *SOX30*, *Pax3*, *OCT-4*, and *Nothc1/2*).

#### 2.3.3. Western Blot Analysis

Collected cells were sonicated for few seconds at 4 °C to extract cellular proteins, and protein concentrations were measured using Bio-Rad reagents. Two µg of total protein from cell lysates were separated with SDS-PAGE gels and transferred to a PVDF membrane (Bio-Rad, Hercules, CA, USA) for 1h in 25 mM Tris-HCl (pH = 8.3), 192 mM Glycine and 20% methanol. Membranes were probed with primary antibody against Keratin 13 ((K13), Santa Cruz Biotechnology, Santa Cruz, CA, USA), Keratin 15 ((K15) Biorbyt, St Louis, MO, USA), F-actin (Invitrogen, Waltham, MA, USA), PCNA (Abcam, Cambridge, MA, USA), Oct4 (MyBiosource, San Diego, CA, USA), Notch1 (Abcam, Cambridge, MA, USA), and Nestin (Thermo fisher Scientific, Waltham, MA, USA). HRP-conjugated secondary antibody was used. Membranes were subjected to Chemiluminescence detection using Luminal according to the manufacturer’s instructions (Amersham Pharmacia Biotech, Piscataway, NJ, USA).

#### 2.3.4. Statistics

Data were obtained from three different biological samples. Bars represent mean values ± SEM. P values were determined via one-way ANOVA and Student–Newman Keuls for multiple group comparisons (Sigma-Stat softdish, San Francisco, CA, USA). Statistical significance was set at *p* ≤ 0.05. Bar graphs were shown as Mean ± SEM, n = 3.

## 3. Results

### 3.1. Oral Mucosa Epithelial Cell Isolation and Expansion

**Figure 1:**
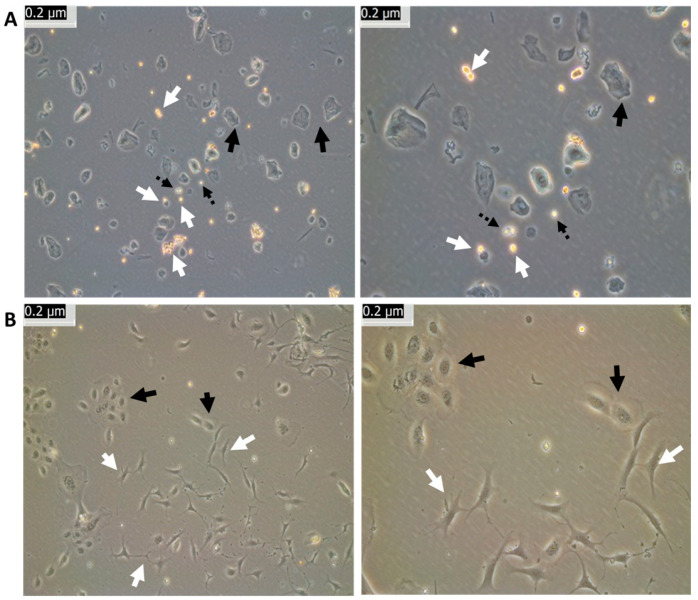
Heterogeneity of oral mucosa epithelial cells. (**A**): Oral mucosa epithelial cells were isolated, seeded, and imaged on day 1, at low (left) and high (right) magnification. (**B**): Oral mucosa epithelial cells were cultured and imaged on day 14, at low (left) and high (right) magnification.

The following experiments were conducted to isolate and expand oral mucosa stem cells with cell culture reagents that were developed for clinical application. Live cell imaging and visual morphological analysis were used to examine cell attachment, confluency, and morphology. Figure 1A (day 1, passage zero (P0)) showed that the isolated epithelial cells were heterogeneous, with different shape and brightness. The polygonal shaped cells (solid black arrows) are mature differentiated epithelial cells, and the small bright cells are “unknown” cells (white arrows). Some of the small cells appeared yellowish (white arrows) and some appeared whitish (black dotted arrows).

After two weeks’ cell culture, the cells’ heterogeneity that was noticed on day 1 was now reduced to only two types of cells (Figure 1B, day 14, P0): epithelial cells (black arrows) and neural-type cells (white arrows).

**Figure 2:**
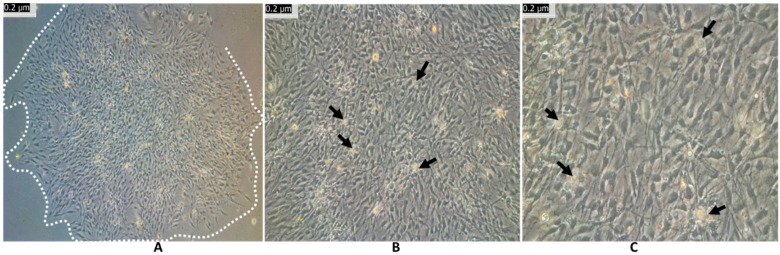
Colony of NCSCs from oral mucosa. On day 22 of cell culture at P0, isolated neural-type cells (**A**) were homogeneous and expanding to form monolayer colonies (**B**,**C**). The dashed line is drawn to indicate the borders of the colony.

After 3 weeks of cell culture, cells self-assembled into colonies with homogeneous cell morphology. Figure 2A shows a colony of neural-type cells. The dashed line is drawn to indicate the borders of the colony. Figure 2B,C depict a higher magnification of the colony. The colonies expanded and formed a monolayer with several center cells (Figure 2B,C, black arrows). Epithelial polygonal cells perished, detached, and were removed during medium changes.

**Figure 3:**
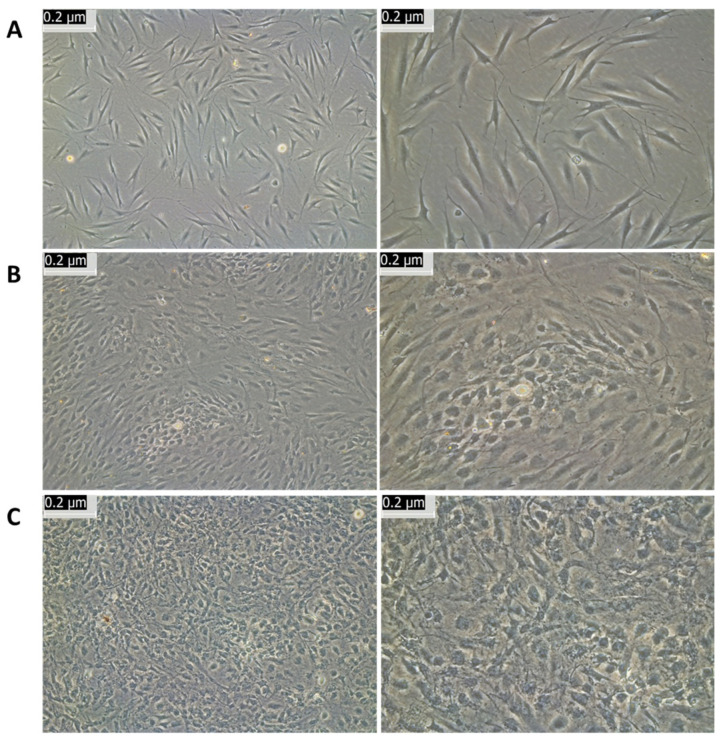
Expansion of NCSCs from oral mucosa. NCSCs were sub-cultured for further expansion. Cells were transferred to passage 1 (P1) and imaged at day 1 (**A**), at day 8 (**B**) and day 24 (**C**) at low (left) and high (right) magnification.

Colonies were then sub-cultured for further expansion. Figure 3 shows the sub-cultured NCSCs at passage 1 (P1) on day 1 (A), day 8 (B), and day 24 (C).

On day 24, the P1 cells appeared similar to the P0 cells on day 22 before passage. The P1 cells grew faster and rapidly formed a monolayer. This indicates that neural-type cells derived from the OM epithelium can be further expanded to build a personalized bank.

### 3.2. Characterization of NCSCs Isolated and Expanded from Oral Mucosa

The characterization of the isolated and expanded NCSCs was conducted to determine and confirm their identification.

Expanded P1 cells were compared to the source-isolated oral mucosa epithelial cells before cell culture. RNA was isolated and analyzed to identify and assess differential expressions. Normalized read count data were used to perform statistical analysis and assess quantitative changes in expression levels between source cells (control) and experimental.

**Figure 4:**
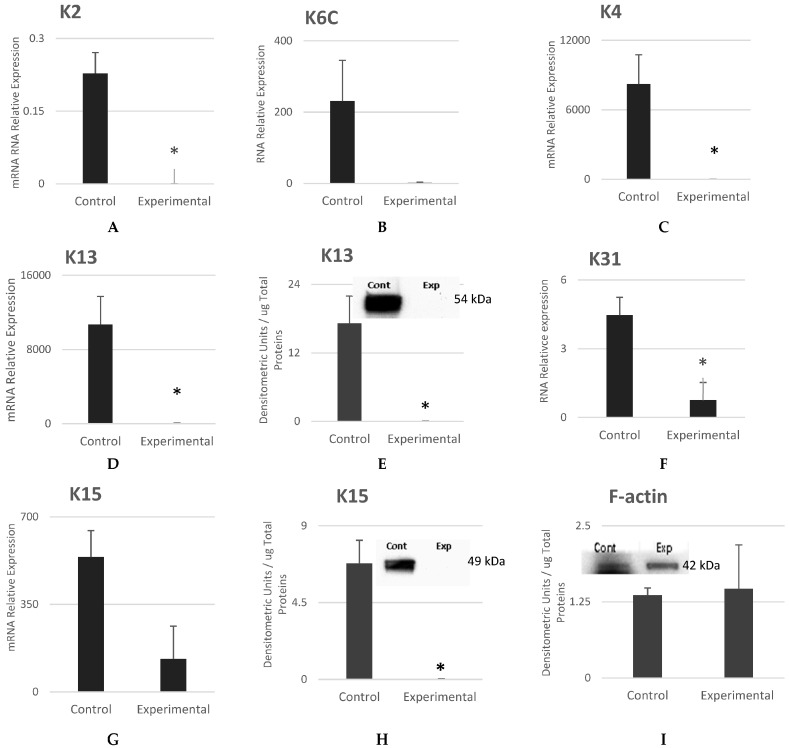
Gene and protein expression analysis of keratins specific to oral mucosa epithelial cells. (**A**–**I**) are the respective expression levels of keratins *K2*, *K6C*, *K4*, *K13*, *K31*, and *K15*. The keratins that are specific to mature differentiated epithelial cells of oral mucosa (control) were not expressed in experimental cells. (**E**) is F-actin used as the loading control. Mean ± SEM, n = 3. The * reflect that the statistical significant difference was achieved.

Figure 4A,B show that mRNA levels of keratin *K2* were expressed with a statistical difference, and those of K6C were expressed with no statistical difference. K13 is the acidic keratin that pairs with its partner, basic keratin (K4), to form the specifically dominant intermediate filaments in epithelial cells of oral mucosa [24]. Figure 4C–E show that the mRNA levels of keratins K4 and K13 were significantly low in the experimental cells. Additionally, K31, which is specific to mature and differentiated epithelial cells, was significantly low in the experimental cells (Figure 4F). K15 is also a specific keratin of oral mucosal epithelial cells and is thought to be a stem cell marker [25,26]. K15 was found to be significantly low in experimental cells when compared to control cells (Figure 4G,H).

We also examined whether experimental cells expressed neural crest stem cell specifiers, such as *Snail*, *SOX*, *Pax3*, *Twist*, and *Hox* genes.

**Figure 5:**
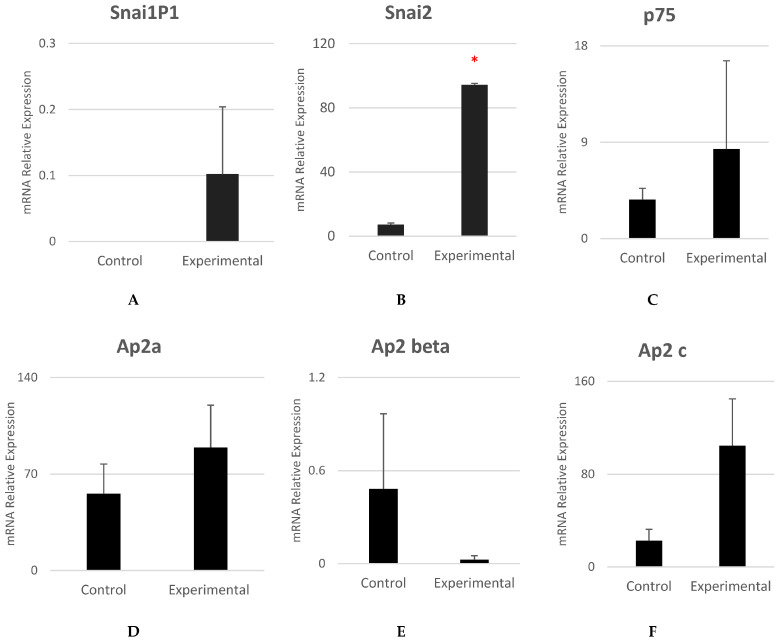
Gene expression analysis of early NCSC specifiers. (**A**–**F**) *SNAI1P1*, *SNAI2*, *P75*, *Ap2a*, *Ap2 beat*, and *Ap2c*. High mRNA expression of *SNAI2* and *Ap2c* was found in experimental cells compared to control cells. Mean ± SEM, n = 3. The * reflect that the statistical significant difference was achieved.

Figure 5A shows that experimental cells expressed the early neural crest specifier *SNAI1P1* with no statistically significant difference. Figure 5B shows that experimental cells expressed the early–mid neurula stage specifier *SNAI2*, and the difference was statistically significant (*p* = 0.031). *SNAI1* gene expression is known to be responsible for inducing the epithelial-to-mesenchymal transition (EMT) and the loss of epithelial markers [27]. *SNAI1P1* is a known pseudogene that has been indicated in influencing a regulatory effect on the expression of *SNAI1* gene.

*P75*, *Ap2a*, and *Ap2c* were found expressed in experimental cells with no statistically significant difference (Figure 5C,D,F). It is possible that *p75* and *Ap2a*, which are highly expressed in NCSCs during development and migration [28,29], were no longer significantly expressed in NCSCs that had reached their target tissue—the buccal mucosa. *Ap2c* has been reported to co-express and form a dimer with *Ap2a* for neural crest induction [30]. It is possible that once NCSCs reach the target tissue, there is no need to express *Ap2a*.

**Figure 6:**
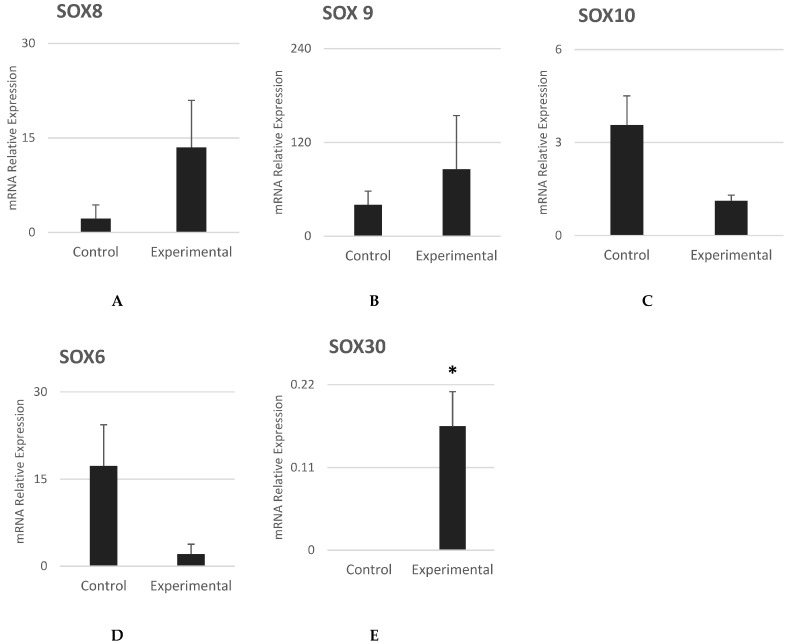
SOX Gene expression analysis of mid-to-late NCSC specifiers of. In experimental cells, mRNA expression of (**A**) *SOX8*, (**B**) *SOX9* and (**E**) *SOX30* was found to be higher than in control cells. However, mRNA expression of (**C**) *SOX10* and (**D**) *SOX6* was found to be lower in experimental cells when compared to control cells. Mean ± SEM, n = 3. The * reflect that the statistical significant difference was achieved.

The earliest neural crest specifiers *SOX8* and *SOX9* were found highly expressed in experimental cells (Figure 6A,B). However, the late neural crest specifier *SOX10* was found to have decreased with no statistically significant difference (Figure 6C). The presence of SRY-box transcription factors (SOX) suggests stem cell maintenance or stem cell differentiation [31]. It has been reported that *Ap2c* was expressed in *SOX10*-knockout human-induced pluripotent stem cells (*SOX10*-/- hiPSCs) [32]. Our results showed a reduced *SOX10* while *Ap2c* was expressed, suggesting that our experimental cells are early-state neural crest stem cells [31].

*SOX6*, which is involved in differentiation into chondrocyte cells [33], was found to be less expressed in NCSCs as compared to control cells with no statistically significant difference (Figure 6D). A possible reason could be an inhibitory mechanism that suppresses its expression in oral mucosa epithelial cells.

*SOX30*, which is required for male fertility in mice [34] and is highly expressed in the testes for sex differentiation [35], was found highly expressed in cells isolated from both males and females, regardless of gender (Figure 6E).

**Figure 7:**
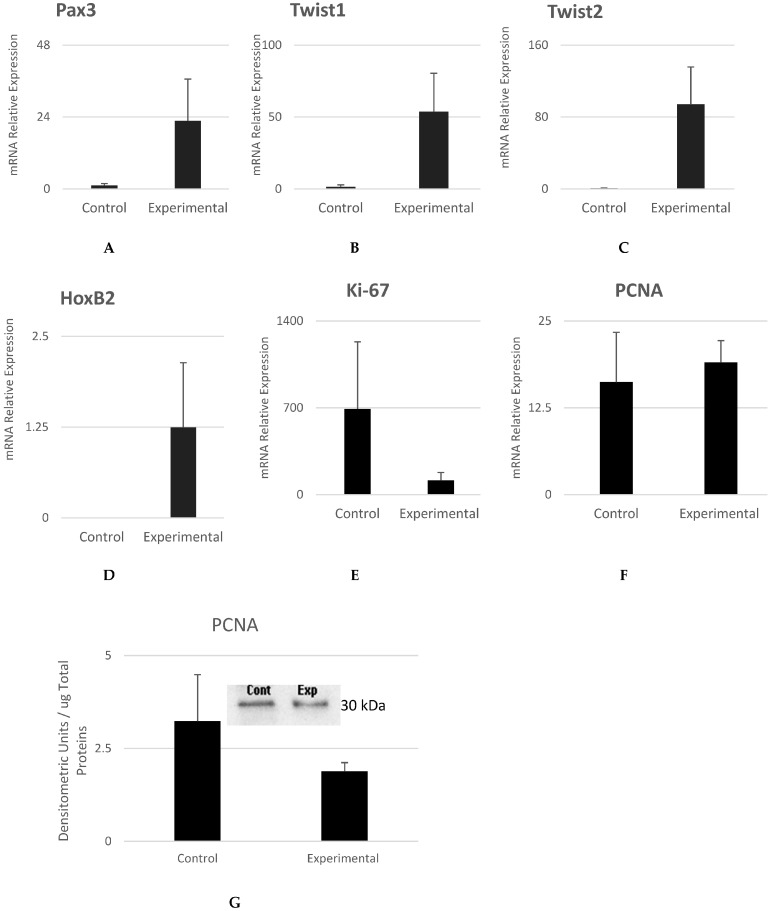
Gene expression analysis of NCSC specifiers responsible for migration to craniofacial tissues. mRNA expression levels of *Pax3*, *Twist1*, *Twist2*, and *HoxB2* were found to be higher in experimental cells as compared to control cells. Gene and protein expression analysis of proliferation markers were also conducted. mRNA expression of *Ki-67* was found to be low as compared to the control. PCNA mRNA and protein levels were similar in control and experimental cells. Loading control is shown in Figure 4 via the measurement of F-actin levels Mean ± SEM, n = 3.

Transcription factor Pax3 plays a major role in the development of multiple neural crest-derived tissues [36,37]. The *Pax3* gene variant has been linked to congenital orofacial defect [38], and its reduced expression has been associated with Sjögren’s syndrome [39]. Our results show that there was a trend in the increased expression of *Pax3* in the experimental cells as compared to control cells (Figure 7A). The expression of *Pax3* indicates that the cells were of neural crest origin. It is not surprising to detect *Pax3* expression in oral mucosa epithelium, as *Pax3* is among the earliest genes involved in defining the identity and fate of neural crest cells that migrate to form or populate the craniofacial tissue.

The *Twist* family of transcription factors are also well known as neural crest specifiers. They are involved in embryonic development [40,41] and regulate the migratory behavior of neural crest cells [42]. Our results show higher expression levels of *Twist 1* and *Twist 2* in experimental cells as compared to control cells with no statistically significant difference (Figure 7B,C). Along with Hoxa2, the transcription factor Hoxb2 is especially specific to the development of neural crest derived from head and jaw tissue [43].

Our results show higher expression levels of Hox2b in experimental cells as compared to control cells with no statistically significant difference (Figure 7D).

mRNA levels of Ki-67 were found to be low in the experimental cells (Figure 7E), and those of PCNA were highly similar between control and experimental cells (Figure 7F,G). We noticed that these cells stopped dividing once they reached 100% confluence, which was confirmed by the results of Ki-67 and PCNA. The cells seemed to have exited the cell cycle and were in a dormant state at the time of confluence, highly similar to when they have reached target tissue [44].

**Figure 8:**
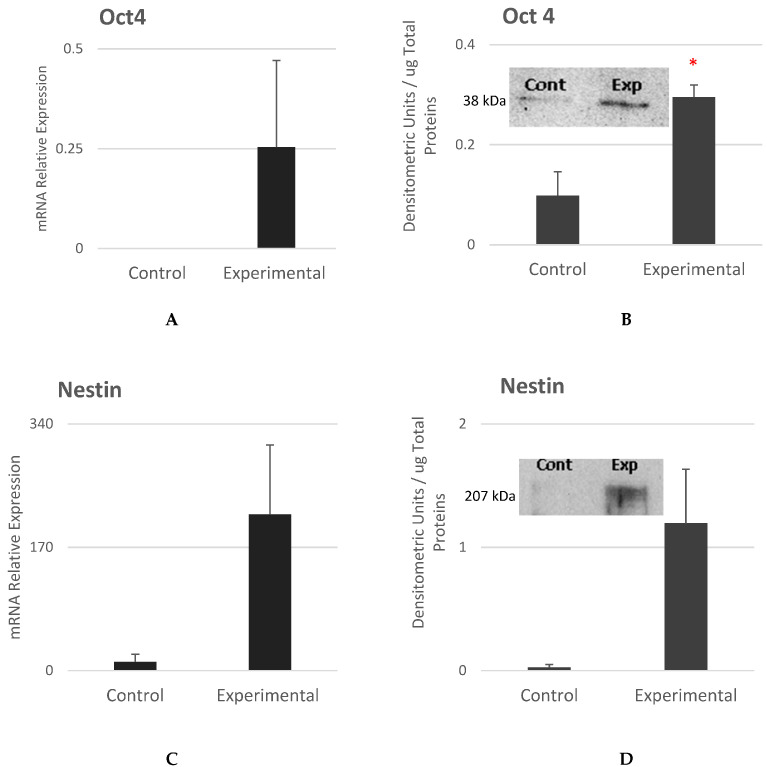

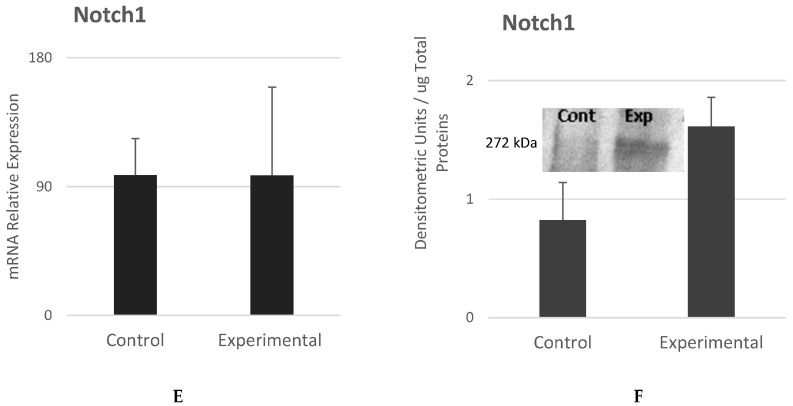
Gene and protein expression analysis of potency and neural progenitor cells markers as compared to the control cells. mRNA expression and protein levels were measured for *Oct4*, *Nestin* and *Notch1*. The loading control is shown in Figure 4 by the measurements of F-actin levels. (Mean ± SEM, n = 3). The * reflect that the statistical significant difference was achieved.

In Figure 8A–F, *Oct4*, *Nestin*, and *Nothc1* were studied to measure the levels of proteins that reflect multipotency of the experimental cells. Oct4 is a pluripotency marker and is involved in the developmental activities of NCSCs [17]. Figure 8A,B show that both *Oct4* mRNA and proteins levels were significantly expressed in experimental cells as compared to control cells.

Nestin is a neuroectodermal stem cell marker that is predominantly detected in neural crest-derived stem/progenitor cells in the growing central nervous system [45,46]. Figure 8C,D show that *Nestin* expression levels were higher in the experimental cells as compared to control cells with no statistically significant difference.

Notch signaling is known to be involved in the developmental processes and maintenance of stem cells [47]. Figure 8E,F show that Notch1 mRNA expression was similar in control and experimental cells. The expression levels of Notch1 protein were found to be higher in the experimental cells as compared to control cells. The statistical difference was not significant.

## 4. Discussion

### 4.1. Neural Crest Stem Cell

During biological development, craniofacial tissues are derived from the differentiation of neural crest stem cells (NCSCs) that migrate from neural border and differentiate into ectoderm and mesoderm [48]. Oral mucosa tissues comprise NCSCs in a quiescent dormant state that changes to an active state when there is a need for adult tissue maintenance, repair, and regeneration via self-renewal and differentiation of tissue-specific cell types [6]. The populations of NCSCs often found in the lamina propria of the oral mucosa tissue can be efficiently differentiated into mesoderm and endoderm cells [6,49]. In the present study, we isolated NCSCs from the epithelium of buccal oral mucosa and were able to expand them using only clinical-grade reagents to demonstrate capability for future clinical applications. When cells were isolated from the epithelium, they were heterogeneous with the dominant typical epithelial cell morphology of polygonal cells. A microscopic examination showed a subset of isolated cells, with very small yellowish and whitish bright cells that were isolated along with the epithelial, connective tissue, and fibroblast cells. After two weeks in culture, cell heterogeneity was reduced to two types of cells, the epithelial cells and the neural-type cells. The results indicated that the reagents used to culture the cells favored the growth and expansion of these two types of cells. However, after 3 weeks, only neural-type lineage-specific cells were found.

### 4.2. Autologous Oral Mucosa Epithelium and Clinical-Grade Reagents for the Isolation and Expansion of NCSCs

There are several limitations and challenges associated with the current methods of isolating and expanding NCSCs. Using embryos or fetal tissue as a source raises ethical concerns. Allogeneic donated NCSCs pose the risks of immune rejection, and immunosuppressive medication may be needed. Access to and supply of NCSCs from adult tissues (dental pulp, hair follicles, and peripheral nerves) without an invasive procedure are very limited. Finally, the regulatory challenges related to the safety of grafted NCSCs represents a major challenge, as no patient should receive cells grown with animal origin reagents. We believe that our cell culture KaFa medium used in these experiments played a significant role in promoting the growth of the neural-type cells. We developed GMP-grade KaFa medium to produce epithelial cell sheets that can be safely grafted back onto patients in clinical applications [18]. The KaFa medium reduces the risk of transmitting any external pathogenic organism that may be a potential source of infection, while improving the consistency of the results by eliminating inherent biological variability due to the use of feeder cells and animal origin reagents. The KaFa medium successfully supported the growth and production of multilayered rabbit oral mucosa epithelial cell sheets [18]. However, when KaFa medium was used to grow human oral mucosa epithelial cells, the results were surprisingly different as after about 3 weeks of culture, only neural-type cells were grown.

At passage 1 (P1), these cells attached to and colonized the surface of the culture ware faster than their predecessor cells at passage zero (P0). It was noticed that once the cells reached 100% confluence, they entered a dormant state, in which they survived for several days without a change of medium. Ultimately, cells were harvested after 3 weeks of culture for characterization and identification.

### 4.3. Genes Expression Specific to Neural Crest Stem Cells

mRNA relative expression of neural crest stem cells specifiers was measured in comparison to source cells isolated on the first day before cell culture. The results showed that the *Snai2* gene, also known as Slug, was significantly expressed in the experimental cells, which is essential to identifying the mesenchymal phenotype of neural cells and their maintenance [50,51]. Deficiency in the expression of *Snai2* was reported to be associated with Waardenburg syndrome type 2 [52]. We also found a high expression of both early transcription factor *SOX8* and *SOX9*, which correlates to the formation of definitive neural crest cells within the neural plate border, indicating that the expanded cells originated from the neural crest border. *SOX30*, which is reported to be required for sex differentiation [35], was interestingly expressed in the experimental cells. This result was surprising, as the biopsies were harvested randomly from both sexes, females and males. *SOX30* may have another essential function in the development of craniofacial tissues.

The expression of *SOX10* was found to be low in the experimental cells, probably because *SOX9* was expressed, causing an inhibitory effect on *SOX10* expression [53]. The expression of *SOX6*, which is required for chondrogenesis, was found to be low in the experimental cells, which is indicative of the fact that oral mucosa tissue does not require cartilage formation [54].

*Pax3* is also a critical regulator of neural crest induction at the neural border, and when mutated, subjects are born with Waardenburg syndrome [55] or with Sjögren’s syndrome [39]. *Pax3* is among the earliest genes expressed in pre-migratory and early migratory neural crest progenitors. It was surprising to detect *Pax3* expression in the oral mucosa cell population. This implies that in adult differentiated oral mucosa epithelial cells, there are early-migrating neural crest stem cells. *Twist* genes are transcriptional factors that induce mesenchymal phenotype similar to the *Snail* gene family. The fact that *Twist* genes are expressed in the experimental cells indicates the non-epithelial feature of these expanded experimental cells. The mechanism for Hox gene expression in a coupling code (Hox code) specific to neural crest cells is not fully understood [43,56]. Our results show that Hoxb2 expression was detected in the experimental cells when compared to the source control cells, confirming the jaw region as the origin of these cells.

### 4.4. Oral Mucosa Epithelium as a Source of Neural Crest Stem Cells

The oral cavity is rich with multiple sources of stem cells, including the mucosa epithelium, the lamina propria, the periodontal ligament, and the dental pulp. The potential of all these stem cells in regenerative medicine is significant. Under normal homeostatic conditions, stem cells are in a quiescent state and are characterized by the expression of specific sets of genes [57]. Some genes are key transcription factors required for their maintenance, and other specifiers are key players in their differentiation into oral mucosa epithelial cells. There are indications of an unknown complex regulation, paracrine signaling, and a synergistic effort among the transcription factors that nudge the quiescent stem cells to enter maintenance or the regenerative state. Despite the upregulation of the transcription factors responsible for EMT induction, the cells grew into a monolayer cell sheet and entered a quiescent and dormant state. The cells did not continue to multiply and proliferate further once they reached 100% confluence. Our cell culture condition supported the growth and expansion of NCSCs but did not change the quiescence of the cells, as they entered a dormant state when they reached complete confluence.

We show that there was no differentiation of oral mucosa epithelial cells into NCSCs. We believe that NCSCs already existed in the mixture of isolated cells and that the KaFa medium supported the isolation and expansion of NCSCs. The isolated and expanded NCSCs from the epithelium of oral mucosa can be differentiated into various human somatic cell types [16].

## 5. Conclusions

Our experiments prove that these cells can be isolated and expanded with clinical-grade medium, ready for differentiation into bone or cartilage cells to repair injuries of the craniofacial tissues, or used in diabetic wound healing, to regenerate corneal epithelium or as a neuroprotection in case of brain injury and neurodegenerative diseases. They can also be used in vitro for personalized disease modeling using the patient’s own cells.

## Data Availability

DNA sequences of study participants will not be shared, as it is not approved by the IRB.
